# Analysis of rs6725887 in the WD Repeat Protein 12 in Association with Coronary Artery Disease in Iranian Patients

**Published:** 2015

**Authors:** Mohammad Piryaei, Sayyed Mohammad Hossein Ghaderian, Hossein Vakili, Hooshang Zaimkohan, Nastaran Mohammadi Ghahhari, Maryam Mafi Golchin

**Affiliations:** 1 *Department of Medical Genetics, School of Medicine, Shahid Beheshti University of Medical Sciences, Tehran, Iran.*; 2 *Cardiovascular Research Center, Shahid Beheshti University of Medical Sciences, Tehran, Iran.*; 3 *Department of Medical Genetics, Faculty of Medicine, Tehran University of Medical Sciences, Tehran, Iran.*; 4 *Department of Biochemistry, Pasteur Institute of Iran, Tehran, Iran.*; 5 *Department of Genetics and Anatomy, Babol University of Medical Sciences, Babol, Iran.*

**Keywords:** Atherosclerosis, *WDR12* gene, polymorphism, risk factor, Iranian

## Abstract

Although genetic variants that affect susceptibility to coronary artery disease (CAD) have been greatly known, a number of these single nucleotide polymorphisms (SNPs) remain to be analyzed in populations with different ethnicities. CAD is influenced by numerous genetic, environmental, and lifestyle factors, and is an important reason for mortality around the globe. In this study, a novel SNP (rs6725887) in the WD Repeat Protein 12 (*WDR12*) gene was selected to be examined in Iranian patients with CAD. Ninety eigth healthy controls and one hundred and one CAD patients were enrolled from Iranian population, and their clinical data were collected for further comparisons. After DNA extraction from each sample, genotypes were characterized by Taq Man probe real- time PCR assay. Statistical analyses were performed to evaluate genotype and allele frequencies and compared the values with clinical variables. Body mass index, blood pressure, fasting blood sugar, LDL, HDL, cholesterol, and triglyceride significantly differed in CAD and control groups. Genotype and allele frequencies of rs6725887 in CAD patients and controls showed no significant association in the distribution. However, clinical parameters of CAD patients like HDL, LDL, FBS, TG, DBP and SBP had significantly (P<0.05) higher levels compared to control group. The rs6725887 polymorphism is unlikely to play a key role in CAD risk in our population. Further additional samples are required for better appreciation of the influence of *WDR12* SNP on CAD occurrence.

Coronary artery disease (CAD) and its main complication, myocardial infarction (MI), are the driving causes of morbidity worldwide. CAD results from atherosclerosis, which is the blockage of major cardiac vessels supplying blood to the heart. The interplay between genetic and environmental determinants and other common risk factors such as cigarette smoking, high blood pressure, diabetes and hypercholesterolemia lead to the pathogenesis of CAD (1-3). Previous studies indicated that genetic factors and biomarkers significantly contribute to the development of CAD. Several comprehensive studies with advanced genome detection techniques such as genome-wide linkage analysis and genome-wide association studies (GWAS) have been successful in providing data on new gene loci that are important in developing CAD (4-6). These techniques may assist scientists to explore and analyze new signaling pathways that are associated with atherosclerosis and the disease involving potential risk (5). Due to differences in geographical or ancestral origin of samples, the association studies encompass different duplicability. Consequently, to improve our understanding about the function of genes in pathology of CAD, apart from variations in ethnicity, genetic and non-genetic factors independent studies should be performed to validate these observations (1, 7).

GWAS of CAD have resulted in the discovery of a novel chromosome locus 2q33.1, being significantly associated with early-onset MI. Kathiresan et al. have recently introduced 2q33 as an MI susceptibility locus that encompasses the* WDR12* gene (8).The gene is approximately 134 kb in length and includes 13 exons and transcribes a member of the WD repeat protein family. WD repeat protein 12 (WDR12) is a ribosome biogenesis protein with 40 amino acids and is formed by gly-his and trp-asp (GH-WD). GH-WD is necessary for proper configuration of heterotri-meric or multiprotein complexes. Biological roles of WDR12 in mammals are yet unknown, but protein is functional in several processes in the cell, such as cell division and proliferation, cell cycle control, and ribosome biogenesis (9). Moreover, it has been suggested that WDR12 is of great importance in neovascularization and regeneration of ischemic tissue and may regulate lipid level (10).

A cluster of single nucleotide polymorphisms (SNP) on 2q33 locus are notably linked to CAD, of which intronic SNP, rs6725887 near *WDR12* gene is significantly associated with early-onset MI (8, 11-14). Therefore, it is an obligation to obtain a comprehensive list of potential disease genes that increase genetic susceptibility to MI and CAD. Likewise because populations of diverse origin show genetic heterogeneity, it is essential to test each risk polymorphism and related genotypes in different ethnic individuals. Generation of a complete picture of susceptible genes in expressing clinically significant phenotypes in different populations remains a challenge for scientists. Most of the large-scale association studies for classifying risk loci in CAD analyzed populations with a different ancestral background exclusive of Iran (15). The present study aimed to examine the association between rs6725887 polymorphism in *WDR12* gene with CAD and various paraclinical indicators in Iranian individuals.

## Materials and methods

**Study population**

The study population comprised of one hundred ninety nine subjects in total consisting of one hundred and one CAD patients diagnosed by positive angiography, and the ninety-eigth remai-ning healthy subjects from both genders. CAD patients underwent angiography to detect luminal narrowing and individuals with stenosis ≥50% were included in the case group. Individuals with negative angiography and cardiac ventriculography were classified as control subjects if they also had no history of MI, hypertension, cigarette smoking, diabetes, obesity, and high level of cholesterol. The study was approved by the local Ethics Committee and Rasearch Council of the Shahid Beheshti University of Medical Sciences. All cases submi-tted signed an informed consent prior to study.

**Analysis of rs6725887 genotype**

Blood samples were obtained and stored in EDTA-containing tubes. Genomic DNA was extr-acted by High Pure PCR Template Preparation Kit (Roche, Germany). DNA was analyzed for quantity and quality by spectrophotometer (Nanodrop 1000, Thermo Fisher Scientific, Wilmington, DE, USA) and using gel electrophoresis. The rs6725887 C>T was genotyped by TaqMan probe real-time PCR. LightCycler 96 (Roche, Germany) was used to determine the genotypes of rs6725887. Primer-probe sets were designed and manufactured by Applied Biosystem custom service.

**Statistical analyzes**

Using SPSS 21 (SPSS Inc, Chicago, IL, USA), clinical data and CAD risk factors such as body mass index (BMI), diabetes, hypertension, LDL, HDL, and triglyceride (TG) were compared between groups by t-test. The chi-square test was utilized to analyze the differences between the qualitative data and groups. One-way ANOVA test was performed to compare the differences in the mean across groups. The association between rs6725887 genotypes and CAD was tested by logistic regression. Stepwise multivariate regression was completed to evaluate the effect of independent variables such as gender and age on genotypes and CAD. Chi-square test was used to test the genotype and allele frequencies for the Hardy-Weinberg equilibrium. A p-value of <0.05 was considered as statistically significant.

## Results

**Clinical characteristics of the study population**

In this study, ninety eight healthy individuals and one hundred and one CAD patients were recruited. The study groups were compared with regard to clinical characteristics and biochemical findings. A detailed description of the clinical parameters and their comparison in the CAD and control groups is shown in Table 1. The presence of hypertension was significantly higher in the CAD group when compared to the controls by T-test (P < 0.0001). The control group showed significantly higher age (61.14 ± 11.05 years) but body mass index (BMI) was higher (27.47 ±6.78) in CAD group compared to those without CAD. Moreover, TG level, and total cholesterol (TC) amount increased considerably in CAD group. Although LDL level was significantly higher in controls, the level of HDL meaningfully increased in the CAD group in comparison to the healthy subjects (P<0.0001).

**Genotype and allele frequencies in the CAD and control groups**

Both genotype and allele frequencies of the rs6725887 C>T polymorphism were in the Hardy- Weinberg equilibrium; however, the genotype and allele frequencies of the SNP were not significantly associated with the increased risk of CAD. There was no difference in the frequencies between CAD and control groups. The genotype distribution of rs6725887 in the control group was CC = 74 (82%), CT = 17 (18%), and TT = 0 (0%). In CAD group, the genotype frequencies of the SNP was 81 (80%) for CC, 17 (18%) for CT, and 3 (2%) for TT. For the allele distributions, the frequencies of the T and C alleles remained constant in both groups (Table 2). Overall, genotype and allele frequencies in the CAD and control groups revealed no significant association in the distribution.

**Table1:** Comparison of clinical characteristics of healthy (control) and angiography negative (CAD) groups

**Characteristics**	**Control (n=98)**	**CAD (n=101)**	**P values**
Age (years)	61.14 ± 11.05	58.65 ± 8.89	0.18
Male	0.49 ± 0.57	0.57 ± 0.49	0.2
BMI (kg/m2)	26.01 ± 4.08	27.47 ± 6.78	0.002
SBP (mm HG)	123.67 ± 14.90	135.81 ± 26.58	<0.0001
DBP (mm HG)	74.35 ± 7.91	83.50 ± 12.75	<0.0001
TG (mg/dl)	116.10 ± 64.71	155.81 ± 68.72	<0.0001
TC (mg/dl)	165.42 ± 27.24	173.57 ± 32.60	<0.0001
FBS (mg/dl)	122.20 ± 39.62	139.05 ± 62.57	<0.0001
HDL (mg/dl)	37.01±5.83	39.16 ± 8.10	<0.0001
LDL (mg/dl)	86.88 ± 2.74	102.04 ± 24.53	<0.0001

Values are represented as mean ± SD for all the variables. BMI: body mass index; SBP: systolic blood pressure; DBP: diastolic blood pressure; TG: triglyceride; TC: total cholesterol; FBS: fasting blood sugar; HDL: high density lipoprotein; LDL: low density lipoprotein

**Table2:** Genotype and allele frequencies for rs6725887 T > C

**OR (95% CI)**	**P-value**	**Control n (91)**	**CAD n (101)**	**Genotype **
1 (Reference)	-	74 (82%)	81 (80%)	**CC**
0.91 (0.42-1.91)	0.81	17 (18%)	17 (18%)	**CT**
6.39 (0.32 to 1.25)	0.22	0 (0%)	3 (2%)	**TT**
1 (Reference)	-	165 (90.66%)	179 (88.61%)	**C allele**
1.24 (0.64 to 2.42)	0.51	17 (9.34%)	23 (11.39%)	**T allele**

**Table3:** One way ANOVA analysis of clinical parameters in association with genotypes

**Variables**	**Control (P value)**	**CAD (P value)**
Age (years)	0.89	0.98
Sex	0.24	0.10
BMI (kg/m^2^)	0.71	0.35
SBP (mm Hg)	0.64	0.90
DBP (mm Hg)	0.19	0.81
TG (mg/dl)	0.72	0.39
TC (mg/dl)	0.21	0.05
FBS (mg/dl)	0.60	0.85
HDL (mg/dl)	0.67	0.18
LDL (mg/dl)	0.84	0.07

BMI: body mass index; SBP: systolic blood pressure; DBP: diastolic blood pressure; TG: triglyceride; TC: total cholesterol; FBS: fasting blood sugar; HDL: high density lipoprotein; LDL: low density lipoprotein.

**Analysis of association between** **rs6725887 genotypes and CAD phenotypes**

We performed a primary analysis for the clinical variables in the control and CAD groups, which showed that age, BMI, systolic blood pressure (SBP), diastolic blood pressure (DBP), and levels of TG, TC, FBS, HDL, and LDL were significantly associated with CAD (Table 1). On the other hand, genotype and allele frequencies were not associated with CAD in the groups (Table 2). To assess whether all these factors were associated with CAD incidence in an independent manner, we conducted one-way ANOVA test but none of the factors were nominated as independent risk factors for CAD in either patient group or control group.

## Discussion

SNPs are among the genetic biomarkers, which could greatly affect the susceptibility of individuals to many diseases (16). Recent advances in polymorphism screening technologies have made possible a more comprehensive type of large scale analysis through GWAS (17). Since CAD is a complex genetic phenotype, it implicates the interaction of a number of genes and environmental elements to determine the incidence of the disease. As of 30 January 2015, 2101 publications and 15268 SNPs discovered to be associated with CAD from GWAS, were included in the catalog of the National Human Genome Research Institute database (18).Description of genes in these studies attempts to collect potential correlated SNPs with CAD, which will benefit the development of personalized medicine in the near future (19-23). However, it should be noted that in addition to their effectiveness in providing detailed information on SNPs, GWASs cannot offer data on all of the polymorphisms in one experiment. The rs6725887 polymorphism in *WDR12* gene has been included in few GWAS analyses, but no report has independently studied this SNP in single population (8, 11, 24, 25). Therefore, our study aimed to investigate this SNP on its intronic locus and replicate the association of rs6725887 with CAD and the correlated risk factors.

In the present study, however, no association between the *WDR12* rs6725887 SNP with the presence of CAD and its risk factors such as hypertension, and levels of FBS, HDL, LDL, and TC could be observed. Although the statistical analysis found a significant difference between the means of variables of CAD risk factors in control and CAD groups (Table 1), none of these elements were associated with the genotype and allele frequencies of the rs6725887 SNP. Furthermore, this study could not detect any significant differences between the genotype and allele frequencies of the SNP in patients and controls in the Iranian population. There is no single study to compare the results of our analysis with; however, GWAS reports on this locus on *WDR12* gene are indicative of an association between the SNP with CAD and MI. For the first time, researchers in Myocardial Infarction Genetics Consortium succeeded in finding the C allele in *WDR12 *rs6725887 as a risk factor in association with CAD and MI (P = 4 × 10 ^-4^, OR = 1.16, 95% CI = 1.10-1.22) in 2,753 samples, but the present study was unable to prove this relationship. In addition, Kathiresan et al. could not reveal the association of this SNP with LDL levels (8).This locus therefore, requires replication in further samples. Later in 2011, O’Donnell etal. performed a meta-analysis of GWASs from 5 cohorts for CAD in 9961 samples with European origin where they found no association between the *WDR12* SNP with CAD/MI (P= 0.09) (11). Therefore, it appears that the results of our study are in accord with those observed by O’Donnell analyses of CAD susceptibility loci. Maouche and Schunkert in 2012 reported the association of 2q33 locus containing rs6725887 with CAD, MI, osteoporosis, and Crohn’s disease (OR= 1.14, 95% CI= 1.09–1.19) (15, 24). In 201, Saade et al. performed a replication study on 2,002 patients and selected nine CAD risk loci including *WDR12* to identify genes predisposing to an increased risk of CAD/MI occurrence. Nevertheless, rs6725887 was not significantly associated with CAD or MI, which is consistent with our results (26). Recently, Blattmann et al. attempted to test the pathogenic effects of genes including *WDR12*, which were previously shown in GWASs to be in association with CAD. They could successfully appoint *WDR12* with a possible role in the regulation of lipid homeostasis as its siRNA-mediated inhibition led to reduced levels of free cholesterol (10). This could suggest the importance of testing candidate genes from GWASs in independent studies with large sample numbers and in people from various ethnic origins.

The main limitation of this study is its small sample size and lack of the functional analysis of the *WDR *gene in our population. In the present study, we analyzed only a single polymorphism but haplotype analysis may better uncover the genetic basis of CAD in our population. Also, the result of the method may be confirmed by using sequencing test.

In conclusion, similar cohort studies with more subjects of both CAD patients and controls will definitely lead to clear results. Additional examination of diverse geographical regions, especially in populations of non-European origin could generalize the results through other ethnic groups.

## Conflict of interests

The authors declared no conflict of interests.
